# 4th International Multithematic Scientific Bio-Medical Congress, Cyprus 2016

**DOI:** 10.1038/cddis.2017.79

**Published:** 2017-03-16

**Authors:** Anastasis Stephanou, Ioannis Patrikios

**Affiliations:** 1 School of Medicine, European University Cyprus, Nicosia, Cyprus

The 4th International Multithematic Scientific Bio-Medical Congress 2016 was held at the European University Cyprus (EUC), Nicosia, Cyprus. EUC is becoming an Institution with high-quality targets aiming to new frontiers of science, innovation, research and excellence. This congress that has now become an annual event with global recognition and subtitled ‘Bio-medical Scientific Cyprus, (BSC)' was founded and established by Professor Dr Ioannis Patrikios, a faculty member of the School of Medicine at EUC.

Tomas Lindah (Nobel Laureate in Chemistry, 2015) gave the keynote presentation with an overview on the intrinsic fragility of DNA. Specifically, he described how DNA in human cells undergoes unavoidable damage, caused by the intracellular reactive oxygen species and other metabolites. He elaborated on how cellular DNA is continuously being repaired and that several DNA repair pathways involved, (including the base excision-repair pathway) is also counteracted occasionally by loss of adenine and/or guanine bases from DNA. His long-standing research contribution provided fundamental insights on DNA replication/repair and has improved our knowledge that will help in development of new therapies like cancer treatments.

Philip Calder spoke on Omega-3 fatty acids and inflammation: from mechanisms to clinical practice. He provided a summary on the anti-inflammatory properties of eicosapentaenoic acid (EPA) and docosahexaenoic acid (DHA) derivatives of omega-3 (n-3) fatty acids found in oily fish and fish oil supplements. He emphasized on the mechanisms underlying the anti-inflammatory actions that include altered cell membrane phospholipid fatty acid composition, disruption of lipid rafts, inhibition of activation of the pro-inflammatory transcription factor nuclear factor kappa B so reducing expression of inflammatory genes, activation of the anti-inflammatory transcription factor peroxisome proliferator activated receptor- and binding to the G protein coupled receptor GPR120. He also documented on human trials that have shown benefits of oral n-3 fatty acids in some inflammatory diseases, such as arthritis and some evidence in a number of other inflammatory diseases.

In the same session, Marios Pantzaris in collaboration with Ioannis Patrikios gave a talk on Neuroaspis plp10, a liquid mixture of specific Polyunsaturated Fatty Acids (PUFAs) together with specific antioxidant vitamins as a holistic treatment model approach for multiple sclerosis (MS). Neuroaspis plp10 has been clinically tested as an adjuvant treatment in MS patients in Cyprus in a double blind phase II clinical trial and has proven its effectiveness by significantly reducing both the clinical relapses and the accumulation of disability. Also, Nikolaos Grigoriadis provided data showing that a possible pathway for controlling MS disease activity might be through targeting B-cells. Among them, rituximab and ocrelizumab, which are typical examples of and-CD20 MAbs for MS. Following on the same subject, Andreas Lysandropoulos provided data suggesting HLA A2, in particular in combination with DRB15, as a marker of better prognosis in MS with respect to Multiple Sclerosis Severity Score and brain volume changes. Finally, the first session was concluded by Theodoros Kyriakides on studies identifying genetic variants for MS that included P-selectin, integrins ITGA4, ITGB1 and ITGB7, adhesion molecules ICAM1, VCAM1 and MADCAM-1, Fibronectin 1 and Osteopontin; with all been shown as possibly be involved in lymphocyte adhesion processes and trafficking to the central nervous system. As it has been suggested, these findings may have implications for prognosis, treatment options and in the selection of potential therapeutic targets.

Gerry Melino open the session on Cancer and new potential treatment approaches and spoke on p53 family members (p63 and p73) in cancer biology. He focused on how these transcription factors are activated (for example during DNA damage) and how such effect is able to modulate mechanisms and regulation of cell death. This was also supplemented with how p53 structure-function relationship such as tetramerization and DNA-binding interactions also controls functional regulation of the p53. Additionally, data was shown that TAp73−/− mice show high tumor incidence and that TAp73 opposes HIF-1 activity by interacting directly with HIF-1a. It was suggested that p73–HIF-1 interaction may be involved in the molecular basis of the growth, progression, and invasiveness of human cancers.

Next, Anastasis Stephanou spoke on the effects of natural phytochemical extracts from Tripterygium wilfordii Hook F, amygdalin and graviola that induced cell death in cancer cells but not in normal cells. Moreover, in an attempt to identify possible targets, an *in silico* approach on the most abundant molecules from the above extracts indicated selective effects on Na+/K+ ATPase and SERCA ATPase channel activity. Furthermore, the effects of these extracts on Na+/K+ ATPase and SERCA ATPase activity were validated. As discussed, such data indicated that these natural phyto-compounds may have distinct targets with reduced drug toxicity for the treatment and prevention of different cancer types.

Finally, Antonis Kirmizis gave a presentation on epigenetics of aging and cancer showing the histone N-terminal acetyltransferase Naa40 as a novel epigenetic regulator of cellular aging. Results indicated that Naa40 activity targets histone H4 and extends cellular lifespan by inducing cellular stress-response pathways in a manner that mimics the effect of calorie restriction. In addition, data showed that colon cancer cells and Naa40 functions as an anti-apoptotic factor and was proposed that such epigenetic enzyme activity may be considered as a therapeutic target.

This, as a meeting report, we have focused on summarizing the major scientific findings from the above-mentioned topics as well as unpublished data.

